# ATF4-mediated transcriptional regulation protects against β-cell loss during endoplasmic reticulum stress in a mouse model

**DOI:** 10.1016/j.molmet.2021.101338

**Published:** 2021-09-20

**Authors:** Keisuke Kitakaze, Miho Oyadomari, Jun Zhang, Yoshimasa Hamada, Yasuhiro Takenouchi, Kazuhito Tsuboi, Mai Inagaki, Masanori Tachikawa, Yoshio Fujitani, Yasuo Okamoto, Seiichi Oyadomari

**Affiliations:** 1 Division of Molecular Biology, Institute for Genome Research, Institute of Advanced Medical Sciences, Tokushima University, Tokushima, 770-8503, Japan; 2 Department of Pharmacology, Kawasaki Medical School, Kurashiki, Okayama, 701-0192, Japan; 3 Fujii Memorial Institute of Medical Sciences, Institute of Advanced Medical Sciences, Tokushima University, Tokushima, 770-8503, Japan; 4 Department of Molecular Research, Diabetes Therapeutics and Research Center, Institute of Advanced Medical Sciences, Tokushima University, Tokushima, 770-8503, Japan; 5 Graduate School of Biomedical Sciences, Tokushima University, Tokushima, 770-8505, Japan; 6 Laboratory of Developmental Biology and Metabolism, Institute for Molecular and Cellular Regulation, Gunma University, Maebashi, Gunma, 371-8512, Japan

**Keywords:** Diabetes, Beta cell, Endoplasmic reticulum stress, Unfolded protein response, Integrated stress response, Activating transcription factor 4

## Abstract

**Objective:**

Activating transcription factor 4 (ATF4) is a transcriptional regulator of the unfolded protein response and integrated stress response (ISR) that promote the restoration of normal endoplasmic reticulum (ER) function. Previous reports demonstrated that dysregulation of the ISR led to development of severe diabetes. However, the contribution of ATF4 to pancreatic β-cells remains poorly understood. In this study, we aimed to analyze the effect of ISR enhancer Sephin1 and ATF4-deficient β-cells to clarify the role of ATF4 in β-cells under ER stress conditions.

**Methods:**

To examine the role of ATF4 *in vivo*, ISR enhancer Sephin1 (5 mg/kg body weight, p.o.) was administered daily for 21 days to Akita mice. We also established β-cell–specific *Atf4* knockout (β*Atf4*-KO) mice that were further crossed with Akita mice. These mice were analyzed for characteristics of diabetes, β-cell function, and morphology of the islets. To identify the downstream factors of ATF4 in β-cells, the islets of β*Atf4*-KO mice were subjected to cDNA microarray analyses. To examine the transcriptional regulation by ATF4, we also performed *in situ* PCR analysis of pancreatic sections from mice and ChIP-qPCR analysis of CT215 β-cells.

**Results:**

Administration of the ISR enhancer Sephin1 improved glucose metabolism in Akita mice. Sephin1 also increased the insulin-immunopositive area and ATF4 expression in the pancreatic islets. Akita/β*Atf4*-KO mice exhibited dramatically exacerbated diabetes, shown by hyperglycemia at an early age, as well as a remarkably short lifespan owing to diabetic ketoacidosis. Moreover, the islets of Akita/β*Atf4*-KO mice presented increased numbers of cells stained for glucagon, somatostatin, and pancreatic polypeptide and increased expression of aldehyde dehydrogenase 1 family member 3, a marker of dedifferentiation. Using microarray analysis, we identified atonal BHLH transcription factor 8 (ATOH8) as a downstream factor of ATF4. Deletion of ATF4 in β-cells showed reduced *Atoh8* expression and increased expression of undifferentiated markers, *Nanog* and *Pou5f1*. *Atoh8* expression was also abolished in the islets of Akita/β*Atf4*-KO mice.

**Conclusions:**

We conclude that transcriptional regulation by ATF4 maintains β-cell identity via ISR modulation. This mechanism provides a promising target for the treatment of diabetes.

## Highlights

• ISR enhancer Sephin1 improved glucose metabolism in diabetic Akita mice.
• β-Cell-specific deletion of *Atf4* promoted β-cell loss in Akita mice.
• Downregulation of ATOH8 enhances the undifferentiation of β-cells.

## Graphical abstract

**Figure:**
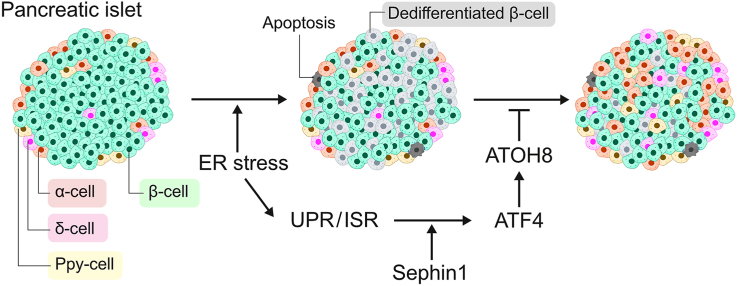

## Abbreviations

4E-BP1: Eukaryotic translation initiation factor 4E-binding protein 1
ALDH1A3: Aldehyde dehydrogenase 1 family member A3
AMPK: AMP-activated protein kinase
ATF: Activating transcription factor
ATOH8: Atonal BHLH transcription factor 8
ChIP: Chromatin immunoprecipitation
CHOP: C/EBP homologous protein
eIF2α: Eukaryotic initiation factor-2α
ER: Endoplasmic reticulum
FoxO1: Forkhead box O1
GO: Gene ontology
HSPA5: Heat shock protein family A member 5
IRE1: Inositol-requiring enzyme 1
ISR: Integrated stress response
Ki67: Antigen KI-67
MANF: Mesencephalic astrocyte-derived neurotrophic factor
mTOR: Mammalian target of rapamycin
Nanog: Homeobox protein NANOG
NKX6.1: Homeobox protein Nkx-6.1
PDX1: Pancreatic and duodenal homeobox 1
PERK: PKR-like ER kinase
POMC: Pro-opiomelanocortin neuron
Pou5f1: POU Class 5 Homeobox 1
PPP1R15A: Protein phosphatase 1 regulatory subunit 15A
RIP: Rat insulin 2 promoter
SIRT1: NAD-dependent protein deacetylase sirtuin-1
TG: Thapsigargin
Tm: Tunicamycin
UPR: Unfolded protein response
WT: Wild-type
XBP1s: Spliced X-box binding protein 1
β*Atf4*-KO: Beta cell–specific *Atf4* knockout

## 1 Introduction

In the prediabetic stage, insulin resistance is compensated by increased insulin secretion that maintains normal blood glucose levels. However, a further increase in insulin resistance exposes β-cells to endoplasmic reticulum (ER) stress because of high insulin demand [[1], [2], [3]]. ER stress is induced in the β-cells of type 2 diabetic animals [4], causing β-cell failure and apoptosis. Furthermore, prolonged ER stress leads to the development of diabetes in several animal models, including Akita insulin-misfolded [5] and Wolfram syndrome mice [6] as well as humans [7, 8].

To adapt to ER stress, cells have specific stress response mechanisms such as unfolded protein response (UPR) [3] and integrated stress response (ISR) [9]. In UPR, three branches involving inositol-requiring enzyme 1 (IRE1), activating transcription factor (ATF) 6, and PKR-like ER kinase (PERK) sense ER stress and maintain ER homeostasis (Supplementary Figure 1). Knockout experiments have shown that IRE1 and PERK regulate proinsulin synthesis and trafficking in the ER of β-cells [[10], [11], [12]]. On the other hand, in ISR, eukaryotic initiation factor-2α (eIF2α) kinases, including PERK, sense various stresses [13,14] and enhance eIF2α phosphorylation. The phosphorylated eIF2α then regulates ER proteostasis through the suppression of global translation. Previous reports show that ISR dysregulation causes deterioration of glucose metabolism and the development of severe diabetes [[15], [16], [17]]. In addition, phosphorylated eIF2α induces several transcriptional factors, including ATF4, raising the likelihood of transcriptional regulation playing an important role in β-cells. ATF4 enhances the mRNA levels of amino acid metabolism- and redox-related genes [18] while inducing the transcription of C/EBP homologous protein (CHOP) that causes ER stress-dependent apoptosis and hyperglycemia. Since the deletion of *Chop* significantly reduces symptoms in several mouse models of diabetes [19,20], the ATF4-CHOP pathway in β-cell could be involved in the pathology of diabetes.

The ISR enhancer Sephin1 has been recently reported to exert curative effects on neurological disorder models, including Charcot-Marie-Tooth 1B and amyotrophic lateral sclerosis [21, 22]. Sephin1 inhibits protein phosphatase 1 regulatory subunit 15A (PPP1R15A), an eIF2α phosphatase, and sustains translational attenuation caused by the increased levels of phosphorylated eIF2α [23]. The phosphorylated eIF2α enhances ATF4 expression and regulates mRNA levels of various factors, including CHOP and PPP1R15A (Supplementary Figure 1). Therefore, studying ISR modulators could be useful in identifying the role of ATF4 in β-cells. In this study, we determined the therapeutic effect of Sephin1 on a diabetic animal model using Akita mice. We also determined the effects of ATF4-mediated transcriptional regulation on β-cells using β-cell–specific *Atf4* knockout (β*Atf4*-KO) mice. The results indicate the involvement of ATF4 in maintaining β-cell identity.

## 2 Materials and methods

### 2.1 Mice

Mice were managed in the animal facilities at Tokushima University in specific pathogen-free conditions under a 12-hour shift of the light–dark cycle. They were fed with the standard rodent food and water *ad libitum*. Rat insulin 2 promoter (RIP)-driven Cre recombinase [24] was used to delete *Atf4* in a pancreatic β-cell–specific manner. The generations of *Atf4*^flox/+^ (F/+) and *Atf4*^flox/flox^ (F/F) mice (C57BL6 background) are described in Supplementary Figure 2. β*Atf4*-KO (*Atf4*^flox/flox^:*RIP-Cre*^Tg/+^) mice were obtained by crossing F/+ and *RIP-Cre*^Tg/+^ mice (provided by Dr. Mark A Magnuson [Vanderbilt University, Nashville, TN, USA], C57BL6 background) through the generation of *Atf4*^flox/+^:*RIP-Cre*^Tg/+^ mice. F/+ and Akita (*Ins2*^C96Y/^^+^, C57BL6 background) mice were crossed to obtain *Atf4*^flox/flox^:*Ins2*^C96Y/+^ mice that were used as the control through the generation of *Atf4*^flox/+^:*Ins2*^C96Y/+^ mice. *Atf4*^flox/flox^:*RIP-Cre*^Tg/+^:*Ins2*^C96Y/+^ (Akita/β*Atf4*-KO) mice were obtained by crossing *Atf4*^flox/+^:*Ins2*^C96Y/+^ and *Atf4*^flox/+^:*RIP-Cre*^Tg/+^ mice. Male mice were used throughout the study because the phenotype is more severe in male Akita mice than in the females. The animal experiments were approved by the Animal Research Committee of Tokushima University, the UK Animals Scientific Procedures Act of 1986, and the EU Directive 2010/63/EU and were performed following the appropriate institutional guidelines.

### 2.2 Cell line

CT215, a mouse pancreatic β-cell line, was established in this study according to the method given for the MIN6-m9 cell line [25] and maintained in DMEM (Nissui Pharmaceutical, Tokyo, Japan) supplemented with 15% (v/v) FBS (Thermo Fisher Scientific, Waltham, MA, USA), 55 μM 2-ME, and nonessential amino acids (Thermo Fisher Scientific—Invitrogen) at 37 °C in a humidified incubator flushed continuously with a mixture of 5% CO_2_ and 95% air. All cell lines were confirmed to be mycoplasma-free.

### 2.3 Plasmid construction and lentiviral preparation

cDNA sequences of human ATF4 and mouse ATOH8 with a C-terminal Myc-DDK tag were cloned into the 3rd generation lentiviral backbone vector containing a hygromycin resistant gene. The DNA sequence of each construct was confirmed using the ABI 3130 DNA sequencer (Thermo Fisher Scientific—Applied Biosystems). The lenti-Cas9-blast vector was gifted by Dr. Feng Zhang (Addgene plasmid #52962). HEK293T cells (ATCC, Manassas, VA, USA) were co-transfected with pMD2.G and psPAX2 (gifts from Dr. Didier Trono [Addgene plasmids #12259 and #12260, respectively]) with each vector using polyethylenimine (Polysciences, Warrington, PA, USA), and conditioned media were then collected. The CT215 β-cells were transduced with each obtained lentivirus using 8 μg/mL hexadimethrine bromide (Sigma–Aldrich, St. Louis, MO, USA) and then selected with hygromycin B (GoldBio, St. Louis, MO, USA) or blasticidin S (KAKEN pharmaceutical, Tokyo, Japan).

### 2.4 Immunoblot analysis

Immunoblot analysis was performed as described previously [26]. The primary antibodies used are listed in Supplementary Table 1.

### 2.5 RNA isolation and quantitative PCR (qPCR)

CT215 β-cells were treated with 200 nM TG (Cayman Chemical, Ann Arbor, MI, USA) for 2 h in the presence or absence of 50 μM Sephin1 (Sigma–Aldrich). Cells were further incubated without TG for 16 h. DMSO, used for dissolving Sephin1, was included at 0.2% (v/v) throughout the treatment. RNA isolation, cDNA synthesis, and qPCR were performed as described previously [27]. The primers used are listed in Supplementary Table 2. The average C_t_ values were normalized to those of the GAPDH gene to obtain the ΔC_t_ values.

### 2.6 Sephin1 administration

The stock solution of Sephin1 acetate salt (Piramal, Mumbai, India) (1.2 mg/mL) was prepared and kept frozen. The aliquots were thawed on the day of treatment, and Sephin1 (5 mg/kg body weight) or vehicle (distilled water) was orally administered to wild-type (WT) and Akita mice after weaning (3 weeks old) between 9:00 and 11:00 a.m. daily for 21 days using a 1-mL syringe attached to a disposable feeding needle. Pharmacokinetic studies of pancreatic tissues were provided by Drs. Anne Bertolotti and Kim Schneider (MRC Laboratory of Molecular Biology, Cambridge, UK) as described previously [21]. Pharmacokinetic studies of plasma were performed using male mice (C57BL6 background) at the age of 8 weeks. Sephin1 acetate salt was orally administrated to mice at a dose of 5 or 10 mg/kg body weight. Plasma samples were obtained over 0, 2, 4, 8, and 24 h after administration. The plasma sample (30 μL) was mixed with 170 μL of ice-cold 90% (v/v) acetonitrile/0.1% (v/v) formic acid containing pazopanib as an internal standard. Subsequently, the mixture was centrifuged at 10,000 *g* for 10 min at 4 °C and the supernatant was analyzed by liquid chromatography–mass spectrometry (Q Exactive Orbitrap, Thermo Fisher Scientific). Mobile phases A and B consisted of 0.1% (v/v) formic acid in water and 0.1% (v/v) formic acid in acetonitrile, respectively. Chromatographic separation was performed on an Xbridge BEH130C18 (1.0 mm I.D. × 100 mm, 3.5 μm, Waters, Milford, MA, USA) at 35 °C with gradients of mobile phase B: 1% for 0–3 min, 1%–100% for 3–8 min, 100% for 8–10 min, and 1% for 10–15 min, at a flow rate of 0.1 mL/min. Mass spectrometric detection was performed by parallel reaction monitoring with the electrospray ionization positive ion mode, using *m/z* 197.64 (precursor ion)/180.03 (product ion) for Sephin1 and 438.52/357.18 for pazopanib.

### 2.7 Physiological analysis

Mice were fasted overnight (16 h) and orally administered glucose solution (2 g/kg body weight) for the analysis of glucose tolerance. Fed and fasting blood glucose levels were measured through the tail blood using Antsense III (HORIBA, Kyoto, Japan) between 9:00 and 11:00 a.m. The levels of HbA_1c_ in whole blood, glycated serum proteins in serum, and 3-hydroxybutyric acid in plasma were determined using Direct Enzymatic HbA_1c_ Assay (Diazyme Laboratories, Poway, CA, USA), Glycated Serum Protein Assay (Diazyme Laboratories), and Autokit 3-HB (FUJIFILM Wako, Osaka, Japan), respectively, according to the manufacturers' instructions. To determine insulin levels, insulin was extracted from the frozen pancreas using 70% (v/v) ethanol containing 0.18 N HCl. The neutralized samples were quantified using the insulin high-range HTRF kit (PerkinElmer—Cisbio, Waltham, MA, USA) according to the manufacturer's instructions.

### 2.8 Immunohistochemistry

The formalin-fixed pancreatic tissues were dehydrated and embedded in paraffin. 5-μm sections were cut. The sections were deparaffinized and then antigen-retrieved in 10 mM Tris–HCl and 1 mM EDTA (pH 8.8) for 15 min at 95 °C. The sections were incubated in PBS containing 0.1% (v/v) Triton X-100 for 5 min, then incubated in the blocking solution (PBS containing 1% [w/v] BSA and 3% [v/v] normal goat serum with or without M.O.M.™ Mouse IgG Blocking Reagent [Vector Laboratories, Burlingame, CA, USA]) for 60 min at 25 °C. The primary antibody was applied overnight at 4 °C or 25 °C, followed by incubation with a secondary antibody for 60 min at 25 °C and then with Hoechst 33258 for 10 min to stain nuclei. The treated sections were visualized with a BZ-X710 microscope (KEYENCE, Osaka, Japan) and analyzed using a BZ-X Analyzer (KEYENCE). The primary antibodies used are listed in Supplementary Table 1. The anti-Ppy antibody was prepared as reported previously [28].

### 2.9 TUNEL

For TUNEL staining, the ApopTag red *in situ* apoptosis detection kit (Sigma–Aldrich) was used according to the manufacturer's instructions. TUNEL-positive cells were visualized using a BZ-X710 microscope and analyzed using a BZ-X Analyzer.

### 2.10 Microarray

Islets of F/F and β*Atf4*-KO mice were isolated as reported previously [29] using collagenase type IV (LS004188, Worthington Biochemical, Lakewood, NJ, USA). The isolated islets were treated with Tm (0.2 μg/mL, Merck, Darmstadt, Germany) for 16 h, and total RNA was extracted using ReliaPrep™ RNA Miniprep System (Promega, Madison, WI, USA) according to the manufacturer's instructions. All samples were prepared from the pooled islets of the four mice in quadruplicate. RNA quality was determined by Quantus (Promega). cDNA was prepared using Complete Whole Transcription Amplification Kit (Sigma–Aldrich) and NucleoSpin Gel and PCR Clean-up (TaKaRa Bio, Kusatsu, Japan), then labeled with Cy3 using Genomic DNA ULS Labeling Kit (Agilent Technologies, Santa Clara, CA, USA) and Micro Bio-Spin 6 Chromatography Columns (Bio-Rad Laboratories, Hercules, CA, USA). Hybridization solutions were prepared from the labeled cDNA using a Gene Expression Hybridization Kit (Agilent Technologies), then applied to SurePrint G3 Mouse GE Microarray 8 × 60K (Agilent Technologies). The data were analyzed by Subio Platform (Subio, Amami, Japan). The raw signal data were converted to processed signal data using global normalization at the 75 percentile and log2 transformation. The genes with the normalized ratios of > 1.5 were considered upregulated or downregulated. The unpaired *t*-test was used to compare two groups, with a cutoff of *P*-values < 0.05 or < 0.2. The false discovery rate for each gene is described in Supplementary Table S4. Gene ontology (GO) enrichment was performed using a Genomatix Genome Analyzer (Genomatix, München, Germany).

### 2.11 Genome editing using a CRISPR-Cas9 system

The specific guide RNA sequences against mouse *Atf4* cDNA were selected using CRISPR Design Tool (http://crispr.mit.edu/) and then cloned into the pX330A-1x3 multiplex gRNA assembly vector (gift from Dr. Takashi Yamamoto, Addgene plasmid #58767) using the Golden Gate cloning method. The multiplex guide RNA cassette was then replaced with the LentiGuide-Puro vector (gift from Dr. Feng Zhang, Addgene plasmid #52963) and the resultant LentiGuide-m*Atf4*-Puro was used for the transduction of Cas9-expressing CT215 β-cells. After puromycin selection, the clonal cell lines were isolated using the limiting dilution method and sequenced by the ABI 3130 sequencer for confirmation.

### 2.12 Chromatin immunoprecipitation (ChIP)-qPCR

WT CT215 β-cells were treated with Tm (0.2 μg/mL) for 16 h, followed by cross-linking with 1% (w/v) formaldehyde for 10 min and sonication 5 times for 30 s each at 310 W in an ice water bath with Bioruptor (Sonicbio, Samukawa, Japan). Samples were prepared by iDeal ChIP-qPCR kit (Diagenode, Seraing, Belgium) with anti-ATF4 antibody (Cell Signaling Technology, Danvers, MA, USA) or control normal rabbit IgG (Cell Signaling Technology) according to the manufacturer's instructions. Immunoprecipitated DNA was purified through MicroChIP DiaPure columns (Diagenode) and analyzed by qPCR, as described above.

### 2.13 *In situ* RT-PCR

*In situ* RT-PCR analysis was performed as described previously, with a few modifications [30]. Briefly, the formalin-fixed pancreatic tissue sections were deparaffinized and antigen-retrieved with Proteinase K Ready-to-use (Agilent Technologies—Dako) for 15 min at 25 °C. Samples were pretreated with 1 U/μL DNase I (Nippon Gene, Tokyo, Japan) containing 1 U/μL RNase inhibitor (TOYOBO, Osaka, Japan) for 2 h at 37 °C. One-step *in situ* RT-PCR was performed using RT-PCR Quick Master Mix (TOYOBO) in a thermal cycler (Mastercycler, Eppendorf, Hamburg, Germany) with an *in situ* adapter. The final concentrations in the reaction mixture were as follows: 1 × RT-PCR Quick Master Mix, 2.5 mM Mn(OAc)_2_, 0.2 μM forward and reverse primers, 0.2 U/μL RNase inhibitor, and 12 μM digoxigenin-11-dUTP (AAT Bioquest, Sunnyvale, CA, USA). The cDNA was synthesized at 60 °C for 30 min. PCR amplification consisted of an initial denaturation step of 94 °C for 1 min, followed by 30 cycles of denaturation (94 °C, 30 s), annealing (60 °C, 30 s), and extension (72 °C, 1 min). The sections were fixed with PBS containing 4% (w/v) paraformaldehyde for 10 min at 4 °C and washed in 0.1 × standard saline citrate (pH 7.0) and maleic acid buffer (100 mM maleic acid, 150 mM NaCl, pH 7.2). After blocking, the sections were incubated with an anti-digoxigenin-rhodamine antibody (Sigma–Aldrich). The treated sections were visualized using a BZ-X710 microscope and analyzed with a BZ-X Analyzer.

### 2.14 Statistical analysis

The data are expressed as means ± SD or SEM. Statistical analyses were performed using Prism (Ver. 8.42; GraphPad Software, San Diego, CA, USA). The unpaired *t*-test was used to compare two groups, while ANOVA and Tukey tests were used to compare three or more groups. *P*-values < 0.05 were considered statistically significant.

## 3 Results

### 3.1 Anti-diabetic effects of Sephin1

**Figure 1:**
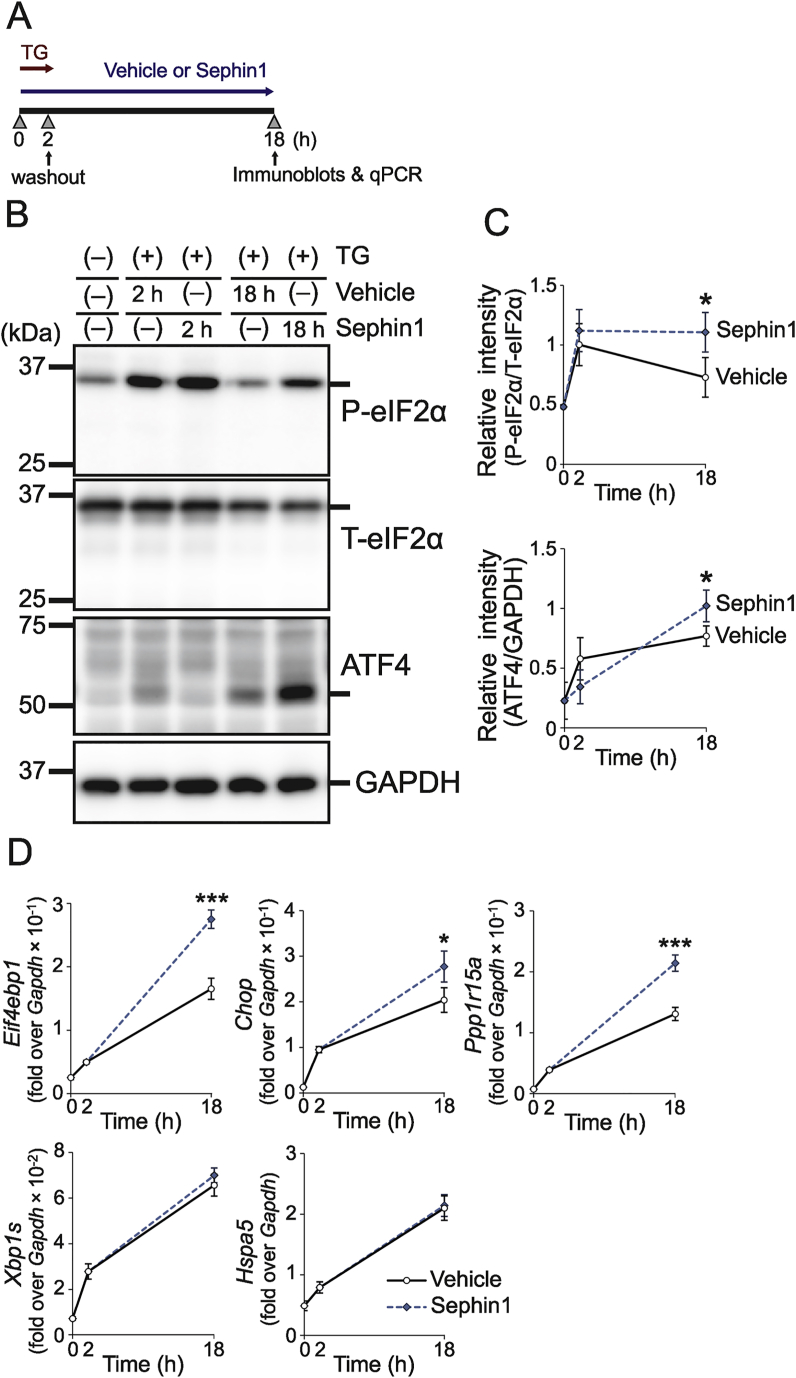
Sephin1 enhances ISR. (A) CT215 β-cells were treated with 200 nM thapsigargin (TG) for 2 h in the presence or absence of 50 μM Sephin1 for 18 h. (B) Representative immunoblots for phosphorylated eIF2α (P-eIF2α) and ATF4 levels in CT215 β-cells. Total eIF2α (T-eIF2α) and GAPDH were used as a loading control. (C) Densitometry quantification of A. Mean ± SD (*n* = 3). (D) mRNA levels of *Eif4ebp1, Chop*, *Ppp1r15a, Xbp1s*, and *Hspa5*. Mean ± SD (*n* = 4). Unpaired *t*-test, ∗*P* < 0.05, ∗∗*P* < 0.01, ∗∗∗*P* < 0.001.

**Figure 2:**
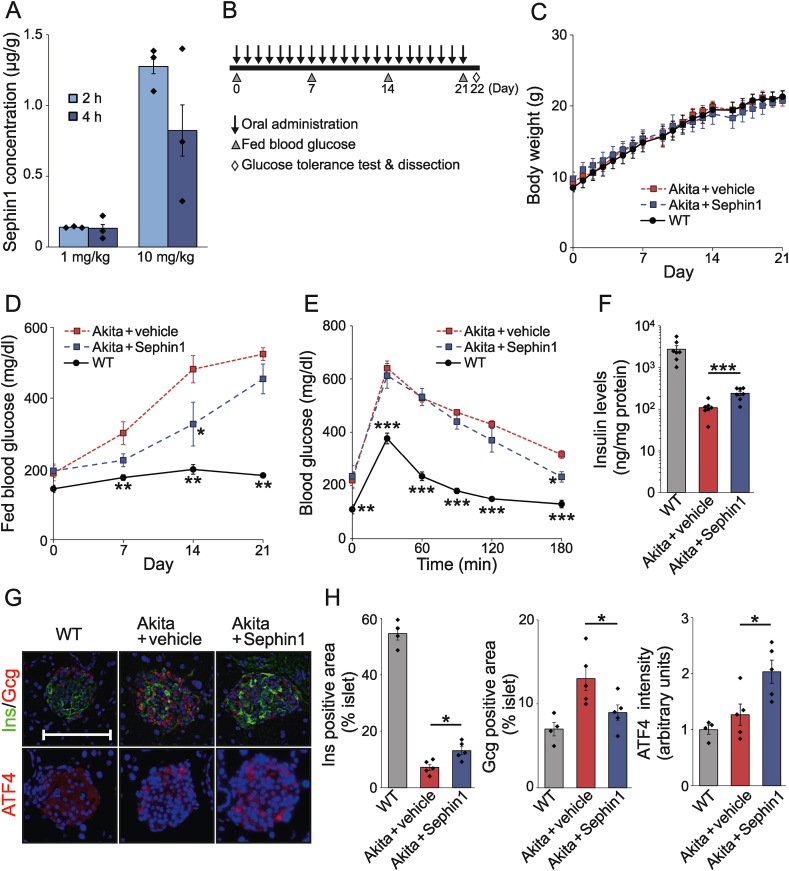
Anti-diabetic effects of Sephin1. (A) Pharmacokinetic analyses of Sephin1. Concentrations of Sephin1 at indicated times after single oral administration of Sephin1 (1 or 10 mg/kg body weight) are shown. Mean ± SEM (*n* = 3). (B) Experimental design of oral administration of Sephin1 to Akita mice. (C) Body weights of WT (*n* = 6) and Akita mice treated with vehicle (*n* = 7) or 5 mg/kg Sephin1 (*n* = 5). Mean ± SEM. (D) Fed blood glucose levels of WT (*n* = 6) and Akita mice treated with vehicle (*n* = 7) or 5 mg/kg Sephin1 (*n* = 5). Mean ± SEM. One-way ANOVA with post hoc Tukey test, ∗*P* < 0.05, ∗∗*P* < 0.01 (vs. vehicle). (E) Oral glucose tolerance test of WT (*n* = 6) and Akita mice treated with vehicle (*n* = 7) or 5 mg/kg Sephin1 (*n* = 5). Mean ± SEM. One-way ANOVA with post hoc Tukey test, ∗*P* < 0.05, ∗∗*P* < 0.01, ∗∗∗*P* < 0.001 (vs. vehicle). (F) Insulin levels of WT and Akita mice treated with vehicle or 5 mg/kg Sephin1. Mean ± SEM (*n* = 7). Unpaired *t*-test, ∗∗∗*P* < 0.001. (G) Representative images of immunofluorescence analysis of pancreatic sections from WT and Akita mice treated with vehicle or Sephin1 showing expression of insulin (Ins), glucagon (Gcg), and ATF4. Blue, nuclei; Green, insulin; Red, proteins (Gcg and ATF4). The scale bar indicates 100 μm. (H) Quantification of relative fluorescence positive areas or relative fluorescence intensity. Mean ± SEM (*n* = 4–5 mice, each containing 5–16 islets). Unpaired *t*-test, ∗*P* < 0.05, ∗∗*P* < 0.01.

First, we examined the effects of Sephin1 on β-cells during ER stress. In CT215 β-cells, thapsigargin (TG) treatment enhanced the protein levels of phosphorylated eIF2α and ATF4 (Figure 1A–C) and the mRNA levels of ATF4 downstream factors, eukaryotic translation initiation factor 4E-binding protein 1 (4E-BP1), CHOP, and PPP1R15A (Figure 1D). The levels of these proteins and mRNAs were enhanced by treatment with Sephin1 for 18 h (Figure 1B–D), while mRNA levels of spliced X-box binding protein 1 (XBP1s) and heat shock protein family A member 5 (HSPA5), ATF4-independent UPR factors, showed no significant difference between the Sephin1-and vehicle-treated cells. Next, we investigated whether Sephin1 was effective in Akita mice. Pharmacokinetic analysis showed a dose-dependent increase in Sephin1 levels in the pancreas and plasma after oral administration (Figure 2A and Supplementary Figure 3), as shown in the previous reports on the brain, sciatic nerve tissues, and plasma [21]. We selected 5 mg/kg as a safe dosage based on previous studies [21,31,32]. Therefore, 5 mg/kg Sephin1 was administered orally to Akita mice once a day for 21 days from 3 weeks of age (Figure 2B), and no significant difference in the body weights and food intakes of Sephin1-and vehicle-treated Akita mice was observed (Figure 2C and Supplementary Figure 4). The deterioration of fed blood glucose levels on day 14 (Figure 2D) and glucose tolerance at the 180-min timepoint on day 22 (Figure 2E) were mitigated by the administration of Sephin1. However, there was no significant difference in fasting blood glucose levels between vehicle- and Sephin1-treated groups (at 0 min in Figure 2E). Sephin1 also enhanced insulin levels (2.37-fold over vehicle treatment, Figure 2F). Furthermore, immunohistochemical analysis showed that the insulin-positive area was decreased in the islets of vehicle-treated Akita mice compared to WT mice. The heterogeneous staining pattern of insulin observed in the islets of the Akita mice might be indicative of accumulation of mutant proinsulin [5]. Sephin1 administration increased the insulin-positive area and decreased the glucagon-positive area in the islets of Akita mice, showing the improvement in β-cell function (Figure 2G,H). Importantly, the intensity of the ATF4 signal was increased by Sephin1 administration (Figure 2G,H). These results showed that ISR enhancement by Sephin1 protects β-cells from loss of function and exerts anti-diabetic effects. This suggests a possibility that these effects are mediated by ATF4.

### 3.2 Diabetes was exacerbated in β*Atf4*-KO mice during ER stress

**Figure 3:**
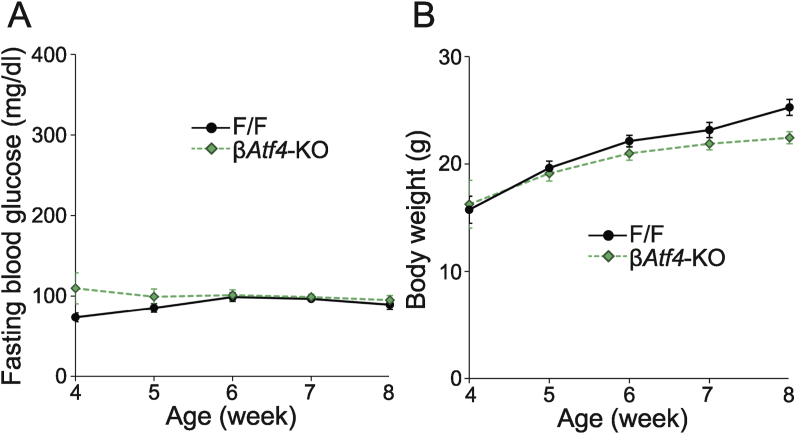
Characterization of β*Atf4*-KO mice. (A) Fasting blood glucose levels of F/F (*n* = 3, 6, 5, 5, and 3 at weeks 4, 5, 6, 7, and 8, respectively) and β*Atf4*-KO mice (*n* = 3, 5, 8, 9, and 11). Mean ± SEM. (B) Body weights of F/F (*n* = 8, 11, 7, 5, and 3) and β*Atf4*-KO mice (*n* = 4, 11, 12, 12, and 14). Mean ± SEM.

**Figure 4:**
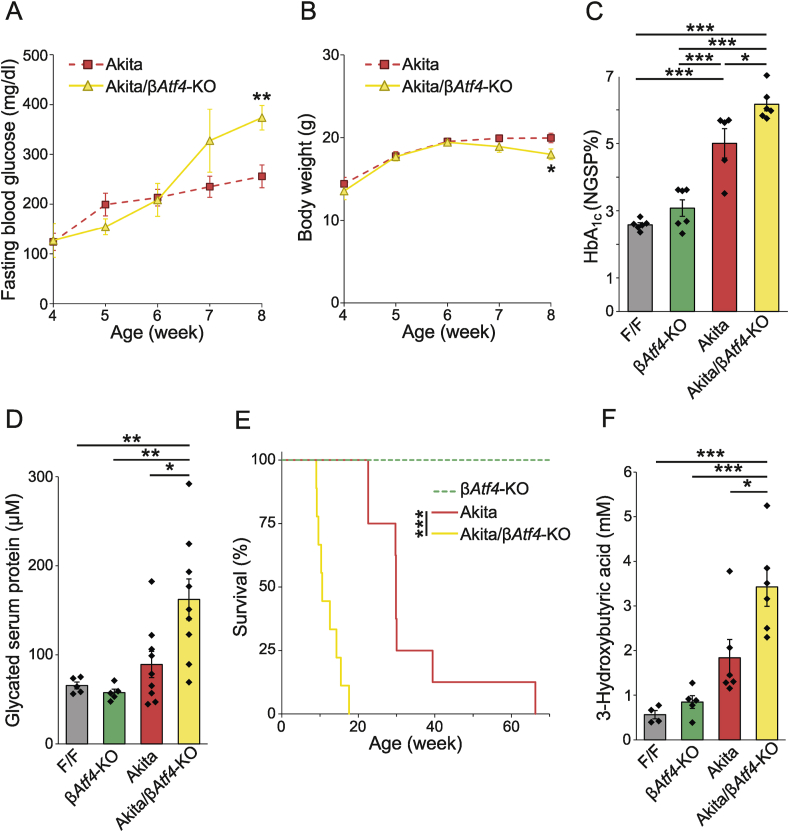
Characterization of Akita/β*Atf4*-KO mice. (A) Fasting blood glucose levels of Akita (*n* = 6, 12, 18, 15, and 11 at weeks 4, 5, 6, 7, and 8, respectively) and Akita/β*Atf4*-KO mice (*n* = 4, 9, 10, 7, and 9). Mean ± SEM. Unpaired *t*-test, ∗∗*P* < 0.01. (B) Body weights of Akita (*n* = 16, 20, 23, 24, and 19) and Akita/β*Atf4*-KO mice (*n* = 11, 15, 13, 14, and 17). Mean ± SEM. Unpaired *t*-test, ∗*P* < 0.05. (C) HbA_1c_ levels in the whole blood of 8-week-old F/F (*n* = 6), β*Atf4*-KO (*n* = 6), Akita (*n* = 5), and Akita/β*Atf4*-KO mice (*n* = 6). Mean ± SEM. One-way ANOVA with post hoc Tukey test, ∗*P* < 0.05, ∗∗*P* < 0.01. (D) Glycated serum protein levels of 8-week-old F/F (*n* = 5), β*Atf4*-KO (*n* = 5), Akita (*n* = 9), and Akita/β*Atf4*-KO mice (*n* = 8). Mean ± SEM. One-way ANOVA with post hoc Tukey test, ∗*P* < 0.05, ∗∗*P* < 0.01. (E) Survival rates in β*Atf4*-KO (*n* = 4), Akita (*n* = 8), and Akita/β*Atf4*-KO mice (*n* = 9). Log-rank test for comparisons between Kaplan–Meier survival curves indicated a significant (∗∗∗*P* < 0.001) increase in the mortality of Akita/β*Atf4*-KO mice compared to that of Akita mice. (F) 3-Hydroxybutyric acid levels in plasma of 8-week-old F/F (*n* = 4), β*Atf4*-KO (*n* = 5), Akita (*n* = 6), and Akita/β*Atf4*-KO mice (*n* = 5). Mean ± SEM. One-way ANOVA with post hoc Tukey test, ∗*P* < 0.05, ∗∗*P* < 0.01.

To investigate the role of transcriptional regulation by ATF4 in β-cells *in vivo*, β*Atf4*-KO mice were developed using mice expressing Cre recombinase under the control of the insulin promoter. Unexpectedly, no significant difference in blood glucose levels (Figure 3A) or body weights (Figure 3B) was observed between F/F control and β*Atf4*-KO mice under normal rearing conditions at the age of 8 weeks. Although we considered the possibility that these mice show a more severe phenotype later on, β*Atf4*-KO mice showed normal blood glucose levels even at 1 year of age (data not shown). Therefore, we crossed β*Atf4*-KO mice with Akita mice to assess the role of ATF4 in β-cells under ER stress conditions. Akita/β*Atf4*-KO mice showed significant hyperglycemia (Figure 4A) and weight loss (Figure 4B) at the age of 8 weeks, compared to those in Akita mice. At this stage, the levels of blood glucose control markers, such as hemoglobin A_1c_ (HbA_1c_) (Figure 4C) and glycated serum proteins (Figure 4D), were significantly higher in Akita/β*Atf4*-KO mice (6.2% ± 0.2% [43.9 ± 2.1 mmol/mol] and 162 ± 23 mM, respectively) than those in Akita mice (5.0% ± 0.4% [31.2 ± 4.8 mmol/mol] and 89 ± 15 mM, respectively). Furthermore, Akita/β*Atf4*-KO mice showed a remarkably shorter lifespan (12.0 ± 1.0 weeks) than Akita mice (33.8 ± 5.0 weeks) (Figure 4E). The plasma concentrations of 3-hydroxybutyric acid in Akita/β*Atf4*-KO mice were 1.9 times higher than those in Akita mice (Figure 4F), suggesting that the shortened life span in Akita/β*Atf4*-KO mice was due to diabetic ketoacidosis.

### 3.3 Loss of β-cells in the islets of Akita/β*Atf4*-KO mice

**Figure 5:**
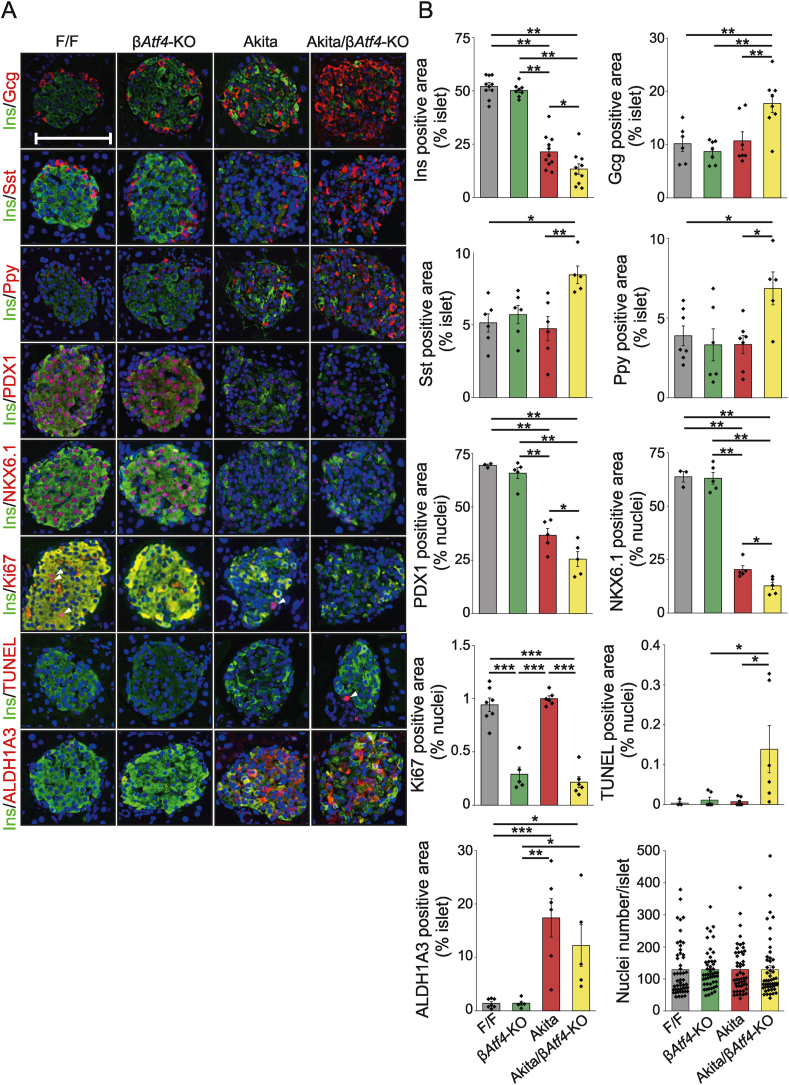
Identification of the cell types of pancreatic islets using immunohistochemistry. (A) Representative images of immunofluorescence analysis from pancreatic sections of 8-week-old F/F, β*Atf4*-KO, Akita, and Akita/β*Atf4*-KO mice showing the presence of insulin (Ins), glucagon (Gcg), somatostatin (Sst), pancreatic polypeptide (Ppy), PDX1, NKX6.1, Ki67, and ALDH1A3. Pancreatic sections from 5-week-old mice were used for TUNEL staining. Blue, nuclei; Green, insulin; Red, indicated proteins. Arrowheads indicate the Ki67-positive or TUNEL-positive nuclei. The scale bar indicates 100 μm. (B) Quantification of relative fluorescence positive areas shown in A (*n* = 3–11 mice, each containing 3–23 islets). The numbers of nuclei per islet were also counted (*n* = 50 islets). Mean ± SEM. One-way ANOVA with post hoc Tukey test, ∗*P* < 0.05, ∗∗*P* < 0.01, ∗∗∗*P* < 0.001.

We investigated the localization patterns of the hormones in the islet using immunohistochemistry (Figure 5A,B). Interestingly, the positive area for insulin was reduced to 62% and the positive area for glucagon, somatostatin, and pancreatic polypeptide (Ppy) was increased by 1.7-, 1.8-, and 2.1-fold, respectively, in Akita/β*Atf4*-KO mice at 8 weeks of age compared to control Akita mice, suggesting that β-cell dedifferentiation is increased by ATF4 deficiency. As expected, the expression of aldehyde dehydrogenase 1 family member A3 (ALDH1A3), a marker of dedifferentiation [33], was significantly increased in Akita and Akita/β*Atf4*-KO mice. There was no significant difference between the total cell numbers in islets of each group, providing additional support for rationale of dedifferentiation. Furthermore, double-positive cells for insulin and glucagon [34] were observed in islets of Akita/β*Atf4*-KO mice (Supplementary Figure 5). The positive areas for pancreatic and duodenal homeobox 1 (PDX1) and homeobox protein Nkx-6.1 (NKX6.1), which are the essential transcription factors for β-cell identity, were reduced to 70% and 63%, respectively, in the Akita/β*Atf4*-KO mice compared to the control Akita mice (Figure 5B). These differences were absent between F/F and *βAtf4*-KO mice. β-Cell proliferation measured using antigen KI-67 (Ki67)-positive area was significantly reduced by ATF4 deficiency in F/F and Akita mice, and the number of apoptotic β-cells measured using TUNEL was significantly increased in Akita/β*Atf4*-KO mice, suggesting that the failure in β-cell turnover was due to ATF4 deficiency.

### 3.4 ATF4 maintains β-cell maturity through ATOH8

**Figure 6:**
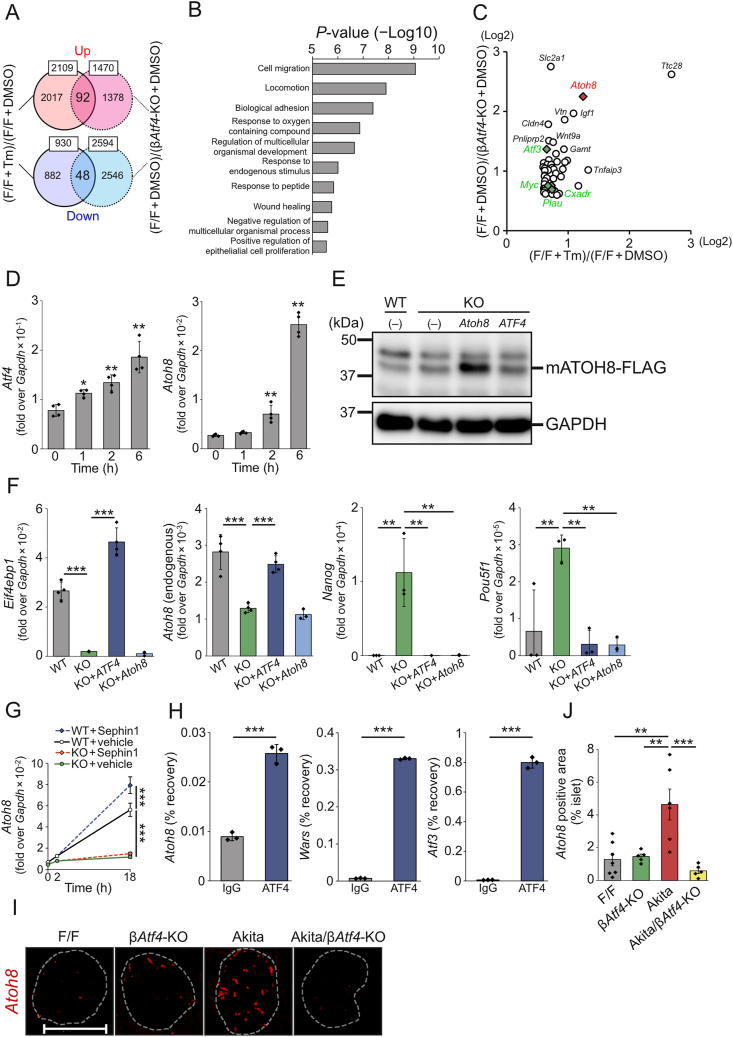
Analysis of ATF4/ATOH8-mediated maintenance of β-cell maturity. (A) Venn diagram shows the overlap of upregulated/downregulated genes in the mouse genome expression profiling microarray datasets ([F/F + Tm]/[F/F + DMSO], *P* < 0.2) and ([F/F + DMSO]/[β*Atf4*-KO + DMSO], *P* < 0.05). (B) Functional classification of the overlapped genes. The overlapping genes (1.5-fold changes) were categorized according to the biological processes using GO annotation. (C) Bubble plot representation of the overlapping genes. Representative hit genes are shown. Previously reported putative ATF4-related genes are shown in red (*Atoh8*) or green (others). (D) mRNA levels of *Atf4* and *Atoh8* in response to 200 nM TG for 0–6 h in CT215 β-cells. Mean ± SD (*n* = 4). One-way ANOVA with post hoc Tukey test, ∗*P* < 0.05, ∗∗*P* < 0.01 (vs. 0 h). (E) Representative immunoblots with CT215 β-cells. WT, wild-type; KO, *Atf4*-KO; *Atoh8*, overexpressing mouse ATOH8-FLAG in the KO cells; *ATF4*, re-expressing human ATF4 in the KO cells. (F) mRNA levels of *Eif4ebp1*, *Atoh8*, *Nanog,* and *Pou5f1* in CT215 β-cells. Mean ± SD (*n* = 3–4). One-way ANOVA with post hoc Tukey test, ∗∗*P* < 0.01, ∗∗∗*P* < 0.001. (G) mRNA levels of *Atoh8* in CT215 β-cells treated with 200 nM TG for 2 h in the presence or absence of 50 μM Sephin1 for 18 h. Mean ± SD (*n* = 4). One-way ANOVA with post hoc Tukey test, ∗∗∗*P* < 0.001. WT, wild-type; KO, *Atf4*-KO. (H) ChIP-qPCR analyses of ATF4 target genes in Tm-treated CT215 cells. Mean ± SD (*n* = 3). Unpaired *t*-test, ∗∗∗*P* < 0.001. (I) Representative images of *in situ* PCR analysis of pancreatic sections from 8-week-old F/F, β*Atf4*-KO, Akita, and Akita/β*Atf4*-KO mice showing the presence of *Atoh8* mRNA. The white dashed lines show the morphology of the islets. The scale bar indicates 100 μm. (J) Relative fluorescence positive areas shown in I. Mean ± SEM (*n* = 5–7 mice, each containing 2–9 islets). One-way ANOVA with post hoc Tukey test, ∗∗*P* < 0.01, ∗∗∗*P* < 0.001.

To identify the downstream factors of ATF4 in the islets, cDNA microarray analyses were performed using the islets of F/F and β*Atf4*-KO mice in the presence or absence of tunicamycin (Tm) treatment. When the vehicle-treated islets from F/F and β*Atf4*-KO mice were compared with a cutoff value of a 1.5-fold change, 1,470 genes were upregulated and 2,594 genes were downregulated out of 55,821 genes in β*Atf4*-KO mice (*P* < 0.05, unpaired *t*-test). Similarly, the Tm treatment of the islets from F/F mice showed upregulation of 2,109 genes and downregulation of 930 genes (*P* < 0.2, unpaired *t*-test). Because the Tm treatment increased the variability, a high *P*-value was used to increase the detection power in GO analysis. It was also notable that the transcriptome profile may have been biased to some extent for technical reasons such as the influence of cells other than β-cells in the islets and whole-transcription amplification from the small amount of islet-derived RNA. Forkhead box O1 (FoxO1) is involved in the differentiation and functional maintenance of the murine β-cells [35]; NAD-dependent protein deacetylase sirtuin-1 (SIRT1) deacetylates and represses the activity of FoxO1 [36]. However, we observed few differences in the expression of these genes between F/F and β*Atf4*-KO mice (Supplementary Table 3). We found that 92 genes were upregulated and 48 genes were downregulated in both of the abovementioned comparisons (Figure 6A and Supplementary Table 4) and focused on upregulated genes to identify the downstream factors of ATF4. We noted that false discovery rates of all genes in Tm treatment of the islets from F/F mice were high (q > 0.184, Supplementary Table 4). Therefore, it is necessary to carefully analyze individual genes. These upregulated genes were associated with the biological processes of the gene ontology (GO) categories, such as cell migration, locomotion, biological adhesion, response to oxygen-containing compound, and regulation of multicellular organismal development (Figure 6B). Among these 92 genes, atonal basic helix-loop-helix transcription factor 8 (ATOH8) has potential implications in the differentiation process during embryonic development [37]. Although ATOH8 regulates the activation of endocrine programs [38], its function in the mature β-cells is unclear. ATOH8 is one of the candidate genes identified as a target of ATF4 using chromatin immunoprecipitation followed by sequencing [39] (Figure 6C). Consistent with this finding, database analysis of mRNA expression in the human pancreas also showed a weak positive correlation between ATF4 and ATOH8 (Supplementary Figure 6). We then determined ATF4-deleted CT215 β-cells using a CRISPR-Cas9 system (Supplementary Figure 7) to establish the relationship between ATF4 and ATOH8 as well as their roles in β-cells during ER stress, because it was technically difficult to isolate morphologically distorted islets from Akita and Akita/β*Atf4*-KO mice. In these cells, decreases in the expressions of ATF4 and its downstream factors (including CHOP, PPP1R15A, and 4E-BP1) were observed (Supplementary Figure 8). We then studied the expression profiles of *Atf4* and *Atoh8* during ER stress in WT CT215 β-cells. The results showed that both mRNA levels were increased after 6 h of TG treatment, but that *Atoh8* was induced slightly later than *Atf4* (Figure 6D), which is consistent with the possibility of ATOH8 being a downstream factor of ATF4. In agreement with this possibility, the ATF4-deleted β-cells showed reduced *Atoh8* expression, while the levels of undifferentiated markers, including homeobox protein NANOG (Nanog) and POU Class 5 Homeobox 1 (Pou5f1, also known as Oct-4), were increased (Figure 6E,F). Furthermore, these increased levels of undifferentiated markers were blunted by ectopic expression of human *ATF4* or mouse *Atoh8*. Notably, TG-induced *Atoh8* expression in WT CT215 β-cells was upregulated by Sephin1 treatment (Figure 6G), which was similar to the expression patterns of ATF4 and its downstream factors indicated in Figure 1C,D. These changes were hardly observed in the ATF4-deleted β-cells, suggesting the uninvolvement of ATF6 and IRE1 pathways. Using ChIP-Atlas (https://chip-atlas.org/), we also examined the upstream sequence of *Atoh8*, which ATF4 was predicted to bind to by ChIP-seq [39], and found the predicted ATF4 binding sites 9.3 kb upstream of the transcription start site of *Atoh8*. We then performed ChIP-qPCR using an antibody directed against endogenous ATF4 in Tm-treated CT215 cells and confirmed binding of ATF4 to the upstream sequence of *Atoh8* as well as to the known ATF4 targets, the promoters of *Wars* and *Atf3* (Figure 6H). Furthermore, we examined *Atoh8* mRNA expression in islets by *in situ* PCR and found that *Atoh8* expression was increased in Akita mice, but was abolished in Akita/β*Atf4*-KO mice (Figure 6I,J). Thus, we propose that ATOH8 regulates β-cell maturity downstream of ATF4.

## 4 Discussion

**Figure 7:**
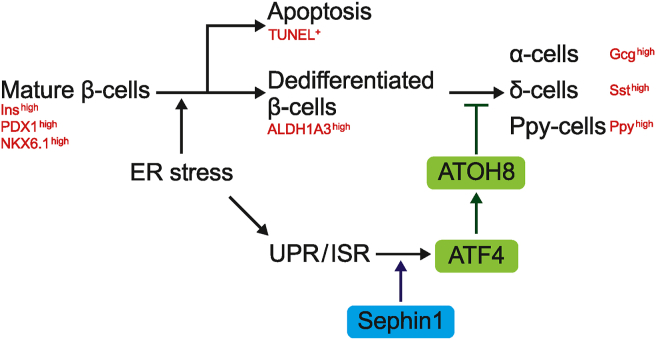
Schematic diagram illustrating the action of ATF4-ATOH8 axis.

In the present study, we found that Sephin1 improved glucose metabolism in diabetic Akita mice. Furthermore, the β-cell–specific deletion of *Atf4* exacerbated diabetes and promoted β-cell loss during ER stress in Akita mice. We also identified ATOH8 as a downstream factor of ATF4 and showed that its downregulation enhances the expression of undifferentiated markers in β-cells (Figure 7). These results promote ISR modulators as potential drugs for the treatment of diabetes. Our results also show the involvement of ATF4 in governing β-cell identity during ER stress.

We observed a significant difference in the fed blood glucose levels between the vehicle- and Sephin1-treated groups on day 14. However, the difference was no longer significant on day 21. It is possible that the accumulation of mutant proinsulin is gradual and cannot be suppressed by Sephin1 on day 21, since the estimated half-lives of Sephin1 in pancreas and plasma were very short (5.2 h and 3.0 h, respectively). Nevertheless, Sephin1 treatment improved insulin levels and increased the insulin-positive area in the islets of Akita mice, suggesting the improvement of β-cell function (Figure 2). ATF4 signals in islets of Akita mice were increased by Sephin1 administration and were on the same level as those in islets of WT in the case of vehicle administration. Since endogenous eIF2α phosphatases (PPP1R15A and PPP1R15B) are highly expressed in β-cells [40], it is suggested that P-eIF2α is promptly dephosphorylated and ATF4 expression is barely elevated in β-cells of Akita mice. In contrast, Sephin1 enhanced ATF4 expression by inhibiting PPP1R15A. Previous studies have reported that glucose metabolism was improved in conditional KO mice lacking *Atf4* in skeletal muscle [41] and liver [42], which are important for insulin sensitivity. If Sephin1 acts on these tissues, then enhanced ATF4 expression in these tissues may lead to insulin resistance. However, it is likely that Sephin1 acts mainly on the pancreas, as glucose metabolism was improved by Sephin1 administration in our experiment (Figure 2). Importantly, Sephin1 does not induce ATF4 on its own and prolongs the expression of ATF4, which is induced by cellular stress such as accumulation of mutant proinsulin. Schneider et al. recently reported that the translation attenuation resulting from eIF2α phosphorylation preferentially targets the mRNAs encoding long-lived proteins and keeps the level of protein synthesis minimal, which is required for protection against various types of stresses [43]. Therefore, it is suggested that Sephin1 increases the phosphorylation of eIF2α to enhance insulin biogenesis by enhancing the folding capacity of ER. However, Sephin1 has been reported to have effects other than the regulation of eIF2α phosphorylation [44,45], and these effects of Sephin1 have not been examined in β*Atf4*-KO mice in the present study. Although our current study indicates that Sephin1 regulates eIF2α phosphorylation through PPP1R15A, other possible mechanisms remain to be elucidated in future studies. On the other hand, chronic stress impairs β-cell identity [46], and the alteration of the identity of β-cells owing to dedifferentiation and/or transdifferentiation is a proposed mechanism for the loss of β-cells in diabetes [35]. Lee et al. recently reported that the deletion of UPR sensor IRE1α induced a transient dedifferentiation of β-cells in non-obese diabetic mice [47]. However, the relationship between dedifferentiation and ISR is poorly understood. We showed that Sephin1 enhanced *Atf4* expression and that targeted deletion of *Atf4* in β-cells of Akita mice (Akita/β*Atf4-*KO) led to an increase in the positive area for glucagon, somatostatin, and Ppy in the islets, suggesting an increase in β-cell dedifferentiation (Figure 4, Figure 5). Therefore, Sephin1 enhances ATF4 to maintain β-cell identity during ER stress. We also showed that the β-cell–specific deletion of *Atf4* significantly reduced β-cell proliferation under normal and ER stress conditions and increased β-cell apoptosis under ER stress conditions (Figure 5). β-Cell turnover is a critical factor in maintaining functional β-cell mass [48]. Recently, the studies using single cells of human islets have shown that the activation of UPR and reduction in insulin production are required for β-cell proliferation [49], and reduced insulin production relieves ER stress conditions and induces β-cell proliferation [50]. A study using β-cell-specific mesencephalic astrocyte-derived neurotrophic factor (MANF)-KO mice showed that MANF is essential for β-cell proliferation [51], and the deletion of *Manf* in the adult mouse β-cells induced ER stress, resulting in β-cell identity loss and death [52]. Interestingly, MANF is also induced by ER stress, suggesting that a certain degree of ER stress is required for β-cell proliferation. Thus, β-cell proliferation and ER stress are considered to be associated with each other. However, further clarification on how ATF4 is involved in β-cell proliferation is required.

ATF4 is ubiquitously expressed and induced by cellular stress. However, some previous studies suggest cell type–dependent effects of ATF4 on metabolism. A report shows that whole-body *Atf4*-KO mice are resistant to age-related and diet-induced obesity because of the reduction in the mammalian target of rapamycin (mTOR) signaling and expression of the gene that regulates the intracellular concentration of amino acids [53]. The liver-specific deletion of *Atf4* in mice suppresses ethanol-induced liver steatosis via the activation of the ATF4/tribbles homolog 3/AMP-activated protein kinase (AMPK) pathway [42]. These results show that ATF4 activates the mTOR pathway (energy excess signal) and inhibits AMPK (energy deficiency signal) during times of increased metabolic demands, leading to impaired metabolic homeostasis. Hypothalamic pro-opiomelanocortin neuron (POMC)–specific *Atf4*-KO mice are resistant to high-fat diet–induced obesity via enhanced autophagy-related 5-dependent autophagy and α-melanocyte–stimulating hormone production, suggesting that ATF4 in POMC neurons negatively regulates energy expenditure [54]. On the other hand, in the present study, β-cell–specific *Atf4* deficiency showed severely compromised β-cell function under ER stress conditions (Figure 4). Our results are similar to those of previous reports showing that the deficiency of the genes induced by ATF4, such as *Eif4ebp1* and *Atf5*, exacerbated hyperglycemia in the mouse models of diabetes [55,56]. Together with the recent observations, our data also indicated the tissue-dependent transcriptional regulation of the expression of ATF4-target genes, which leads to the protection of β-cells from diabetes. In addition, overexpression of *Atf4* compromises postnatal β-cell function [57], suggesting that maintenance of a balanced state or the context-dependence of ATF4-mediated transcriptional regulation are likely important in preserving β-cell function and thereby protection against diabetes. One of the potential compounds that regulate ATF4 expression is Exendin-4 (Exenatide), the first glucagon-like peptide 1 receptor agonist used clinically as a therapeutic agent for type 2 diabetes [58]. Yusta et al. reported that Exendin-4 stimulates ER stress-induced ATF4 expression in β-cells [59]. Thus, our results suggest that ATF4-mediated transcriptional regulation in β-cells may be one of the mechanisms for the anti-diabetic effect of Exendin-4. Further studies using Akita/β*Atf4*-KO mice are needed to determine the extent to which the anti-diabetic effects of Sephin1 and Exendin-4 are dependent on ATF4.

β-Cell identity is known to be regulated by transcription factors such as PDX1, NKX6.1, and FOXO1 [60]. In Akita/β*Atf4*-KO mice, the levels of insulin, PDX1, and NKX6.1 were reduced. Despite the reduced β-cell turnover in the islets in these mice, the number of α-, δ-, and Ppy-cells increased (Figure 5). Furthermore, the expression of the dedifferentiated marker ALDH1A3 [33] was significantly upregulated in Akita and Akita/β*Atf4*-KO mice. The lower ALDH1A3 expression in Akita/β*Atf4*-KO mice compared to Akita mice may be due to the ALDH1A3-negative change in Akita/β*Atf4*-KO mice, as the dedifferentiated β-cells redifferentiated into α-, δ-, and Ppy-cells. We also showed that double-positive cells for insulin and glucagon [34] were observed in the islets of some Akita/β*Atf4*-KO mice. These data suggest the dedifferentiation of mature β-cells in Akita/β*Atf4*-KO mice.

Therefore, the ATF4-mediated pathway is critical for the preservation of β-cell identity, and the failure of this process is associated with the development of diabetes. Although the mechanism of β-cell dedifferentiation is still unclear, it is reported that *Foxo1*-KO mice undergo β-cell dedifferentiation under metabolic stress conditions [35]. Previous studies report that FOXO1 and ATF4 cooperate to regulate glucose homeostasis in osteoblasts [61] and that SIRT1 regulates FOXO1 activity via its deacetylation [36]. However, as shown in Supplementary Figure 8, no remarkable difference in the expression of FoxO1 and SIRT1 was observed between ATF4-deleted and WT β-cells, suggesting that unknown factors are involved in mediating β-cell dedifferentiation by ATF4. Among the comprehensive gene expression profiles, we focused on the transcription factor ATOH8. Previous studies report that ATF4 may directly regulate ATOH8 expression [39]. ATOH8 is expressed in endocrine and exocrine precursor cells in the pancreas [37], and δ-cell number is increased in pancreas-specific *Atoh8*-KO mice [38]. ATOH8 suppresses the transcription of stem cell–related genes such as *NANOG* and *POU5F1* in tumors [62]. Similar to these findings, we observed that *Atoh8* expression was reduced while the expression of undifferentiated markers (*Nanog* and *Pou5f1)* were upregulated in *Atf4*-deleted β-cells during ER stress (Figure 6). Moreover, *in situ* PCR analysis showed that *Atoh8* expression was enhanced in the islets of Akita mice and reduced in the islets of Akita/β*Atf4*-KO mice. ChIP-qPCR using a β-cell line showed that ATF4 bound weakly to the upstream region of *Atoh8*, suggesting that ATF4 is involved in ATOH8 expression. It is also possible that ATF4 forms heterodimers with other transcription factors (e.g. PDX1) [63] and regulates ATOH8 expression. Although our observations partly explain the β-cell dedifferentiation observed in β*Atf4*-KO mice, further studies are required to fully understand the molecular mechanism of the ATF4-dependent β-cell identity. In addition, compounds that can target the downstream factors of ATF4, such as ATOH8, can act as potential therapeutic agents for diabetes.

In conclusion, we showed that transcriptional regulation by ATF4 maintains β-cell identity via ISR modulation. This mechanism therefore provides a promising target for the treatment of diabetes.

## Author contributions

Conceptualization, SO; Formal analysis, KK; Investigation, KK, MO, JZ, YH, YT, MI, and MT; Resources, YF; Data curation, KK, MO, MI, and SO; Writing—Original Draft, KK, KT, YO, and SO; Writing—Review & Editing, all authors; Visualization, KK; Supervision, SO; Project administration, KK and SO; Funding acquisition, KK, YO, and SO. All authors approved the manuscript for publication.
