# Supplementary material for: ATF4-mediated transcriptional regulation protects against β-cell loss during endoplasmic reticulum stress in a mouse model
Source: Mol Metab. 2021 Sep 20;54:101338. doi: 10.1016/j.molmet.2021.101338 (PMC8487982; doi:10.1016/j.molmet.2021.101338)
Supplement: Multimedia component 1 [file mmc1.docx]

**Supplementary data**

**
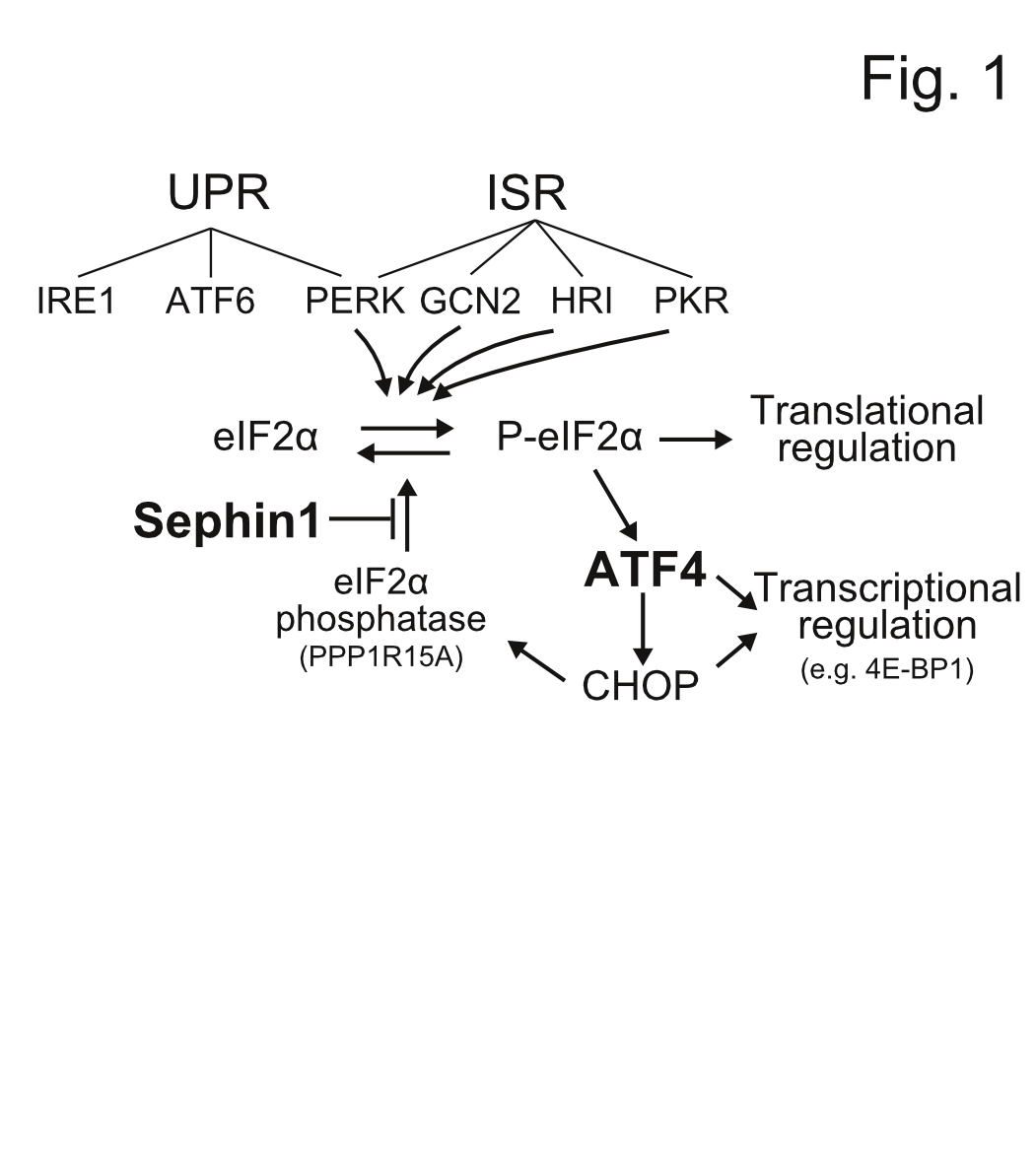
**

**Supplementary Figure 1. Schematic diagram illustrating the action of ISR enhancer Sephin1.**


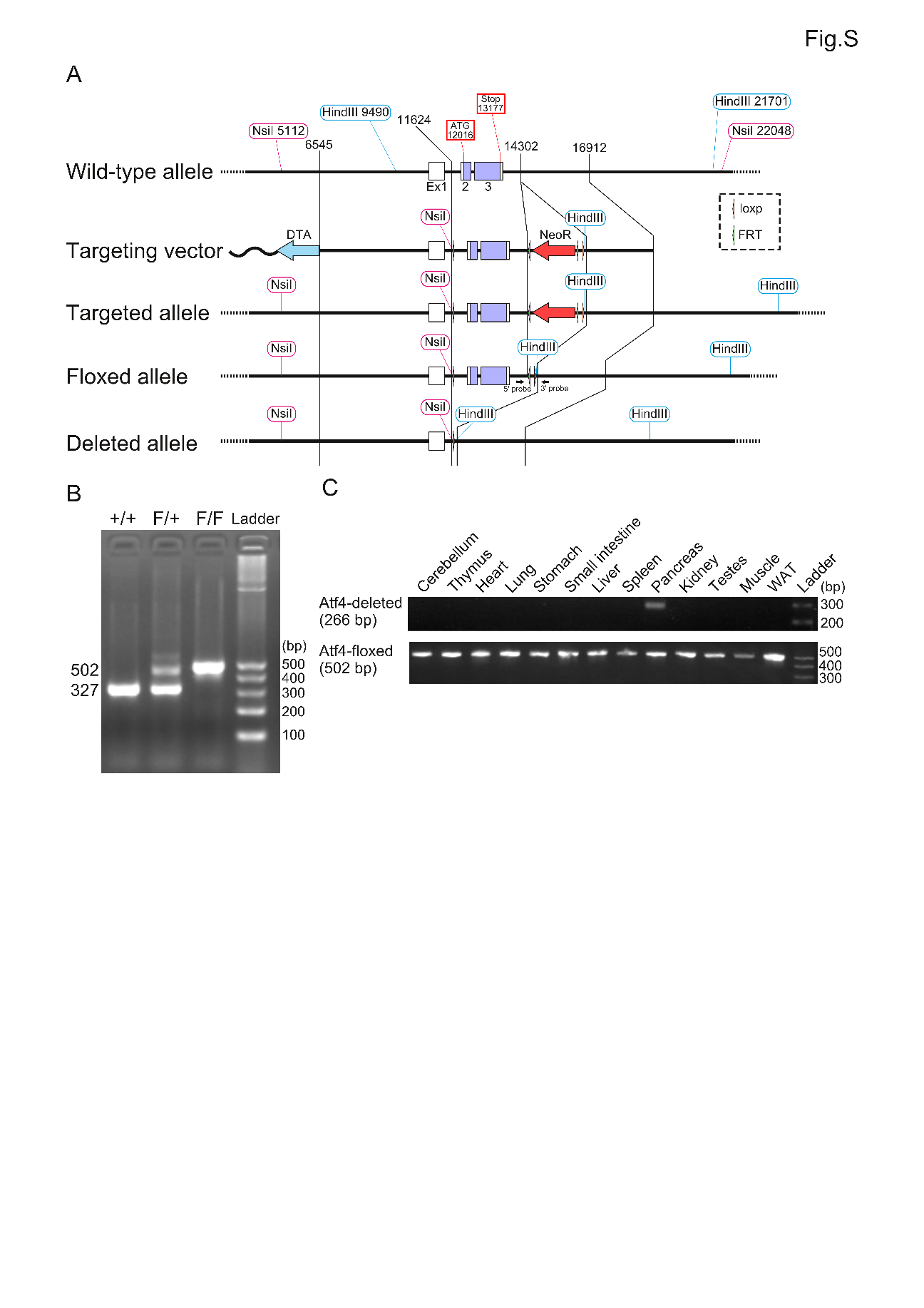


**Supplementary Figure 2. Generation of β-cell-specific *Atf4* knockout (β*Atf4*-KO) mice.**

**(A)** Schema showing the strategy to delete *Atf4*. The introduced cassette and *Atf4* are deleted in the Cre-expressing cells. DTA, diphtheria toxin fragment A; FRT, flippase recognition target. **(B)** Genotyping of mice by PCR. Genomic DNA was extracted from mice of different *Atf4* genotypes and subjected to amplification by PCR. The deduced genotype and product size are shown. **(C)** PCR-genotyping of various tissues of the β*Atf4*-KO mice. The PCR products represent the *Atf4*-deleted allele (266 bp) and floxed allele (502 bp). WAT, white adipose tissue.

**
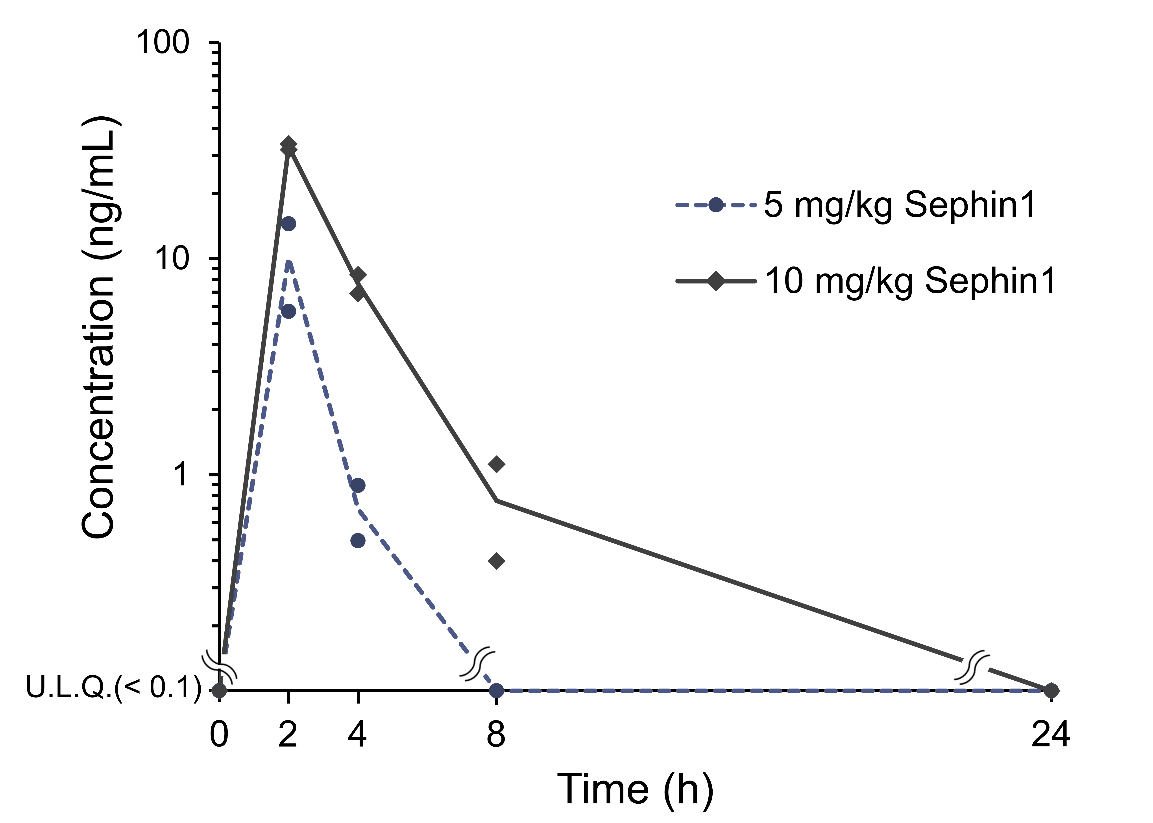
**

**Supplementary Figure 3. Time-course of Sephin1 concentration in plasma.**

Concentrations of Sephin1 in plasma of WT mice were determined over 0, 2, 4, 8, and 24 h after a single oral administration of 5 or 10 mg/kg Sephin1. Each curve represents the mean (*n* = 2 mice). U.L.Q., under the limit of quantification.

**
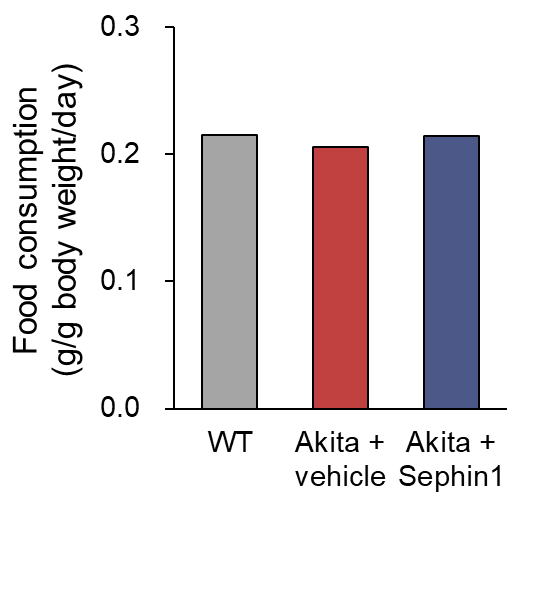
**

**Supplementary Figure 4. Food intake of mice treated with Sephin1.**

Food intake of WT mice (*n* = 2) and Akita mice daily treated with vehicle (*n* = 4) or 5 mg/kg Sephin1 (*n* = 2) from day 1 to day 6. Data are expressed as the mean of 6 days.


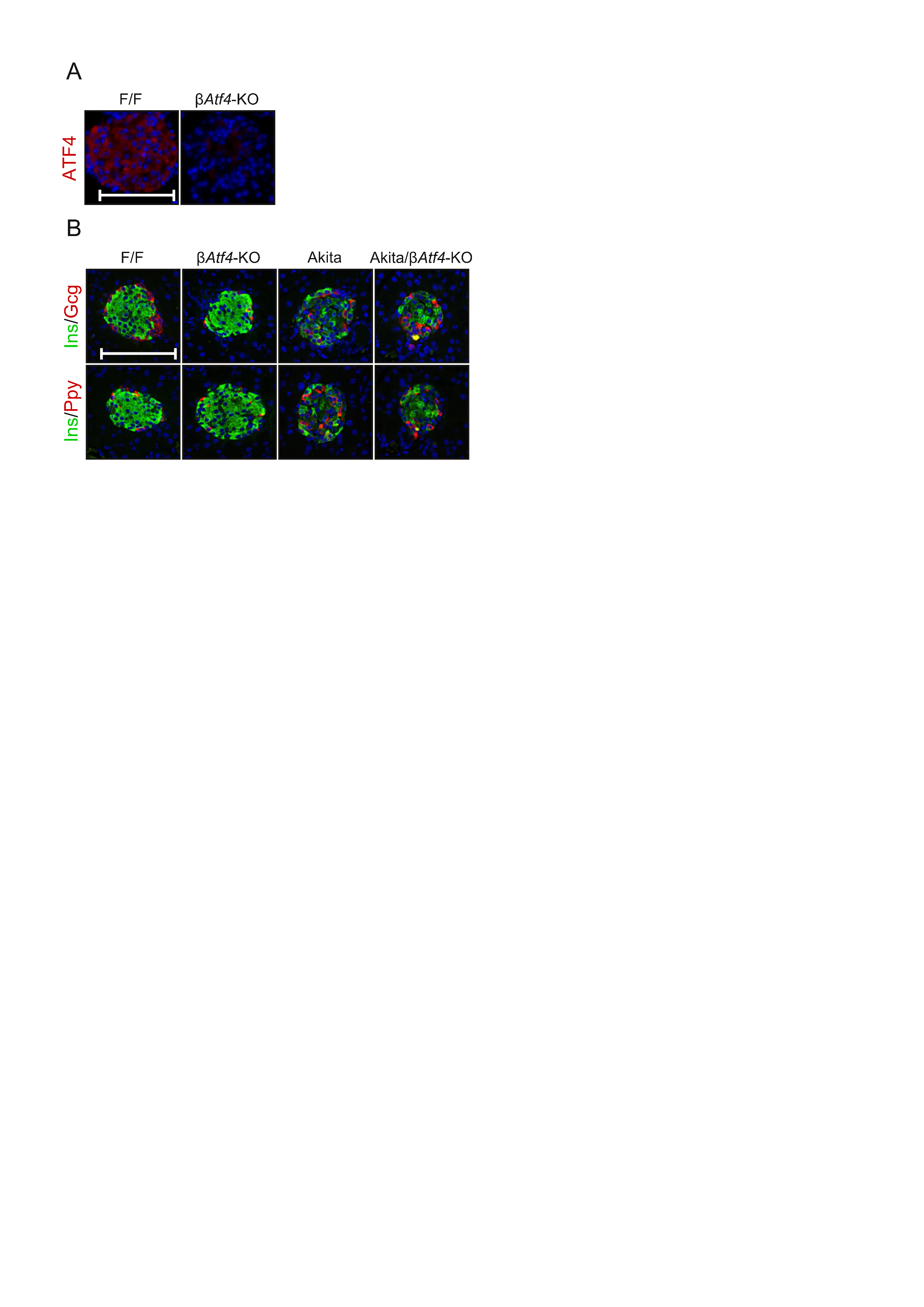


**Supplementary Figure 5. Immunohistochemical analysis of 5-week-old mice.**

Representative images of immunofluorescence analysis of pancreatic sections from F/F, β*Atf4*-KO, Akita and Akita/β*Atf4*-KO mice showing expression of insulin (Ins), glucagon (Gcg) and pancreatic polypeptide (Ppy). Blue, nuclei; green, insulin; red, indicated proteins. Scale bar is 100 μm.

**
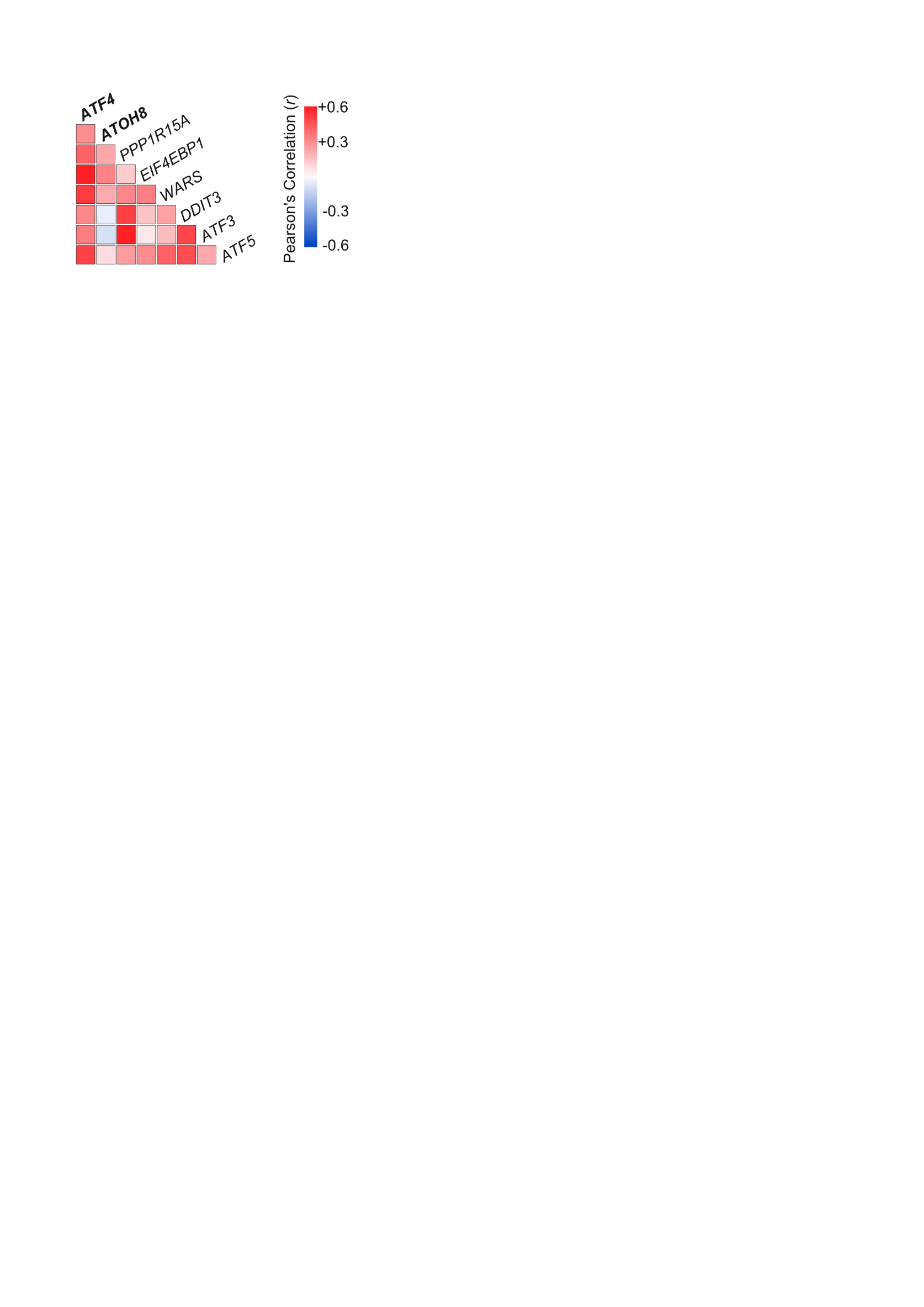
**

**Supplementary Figure 6. Expression of ATOH8 positively correlates with ATF4 and its downstream factors in human pancreas.**

Pearson’s correlation co-expression was calculated using GeneNetwork (www.genenetwork.org). Heat-map showed *ATF4*, *ATOH8*, *PPP1R15A*, *EIF4EBP1*, *WARS*, *DDIT3*, *ATF3* and *ATF5* in pancreas of human genetic reference population. Positive and negative correlations are indicated in red and blue, respectively. The intensity of the colours corresponds to correlation coefficients.


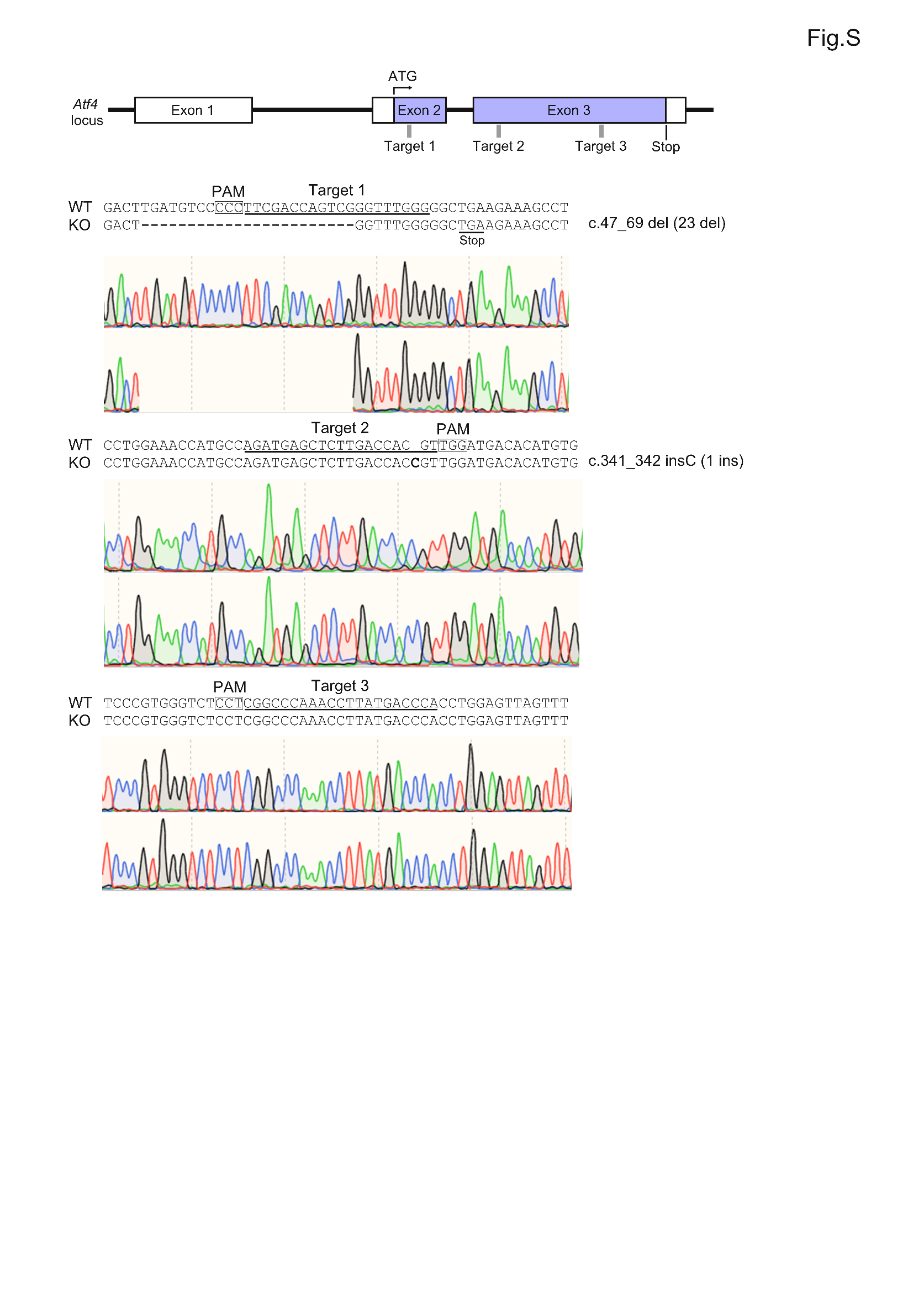


**Supplementary Figure 7. Sequence analysis of genomic DNA extracted from *Atf4* knockout cell line.**

A vector expressing three types of gRNA targeting the *Atf4* sequence was introduced into CT215 β-cells expressing Cas9. Direct sequencing was performed after amplification of genomic DNA by PCR. PAM, protospacer adjacent motif.

**
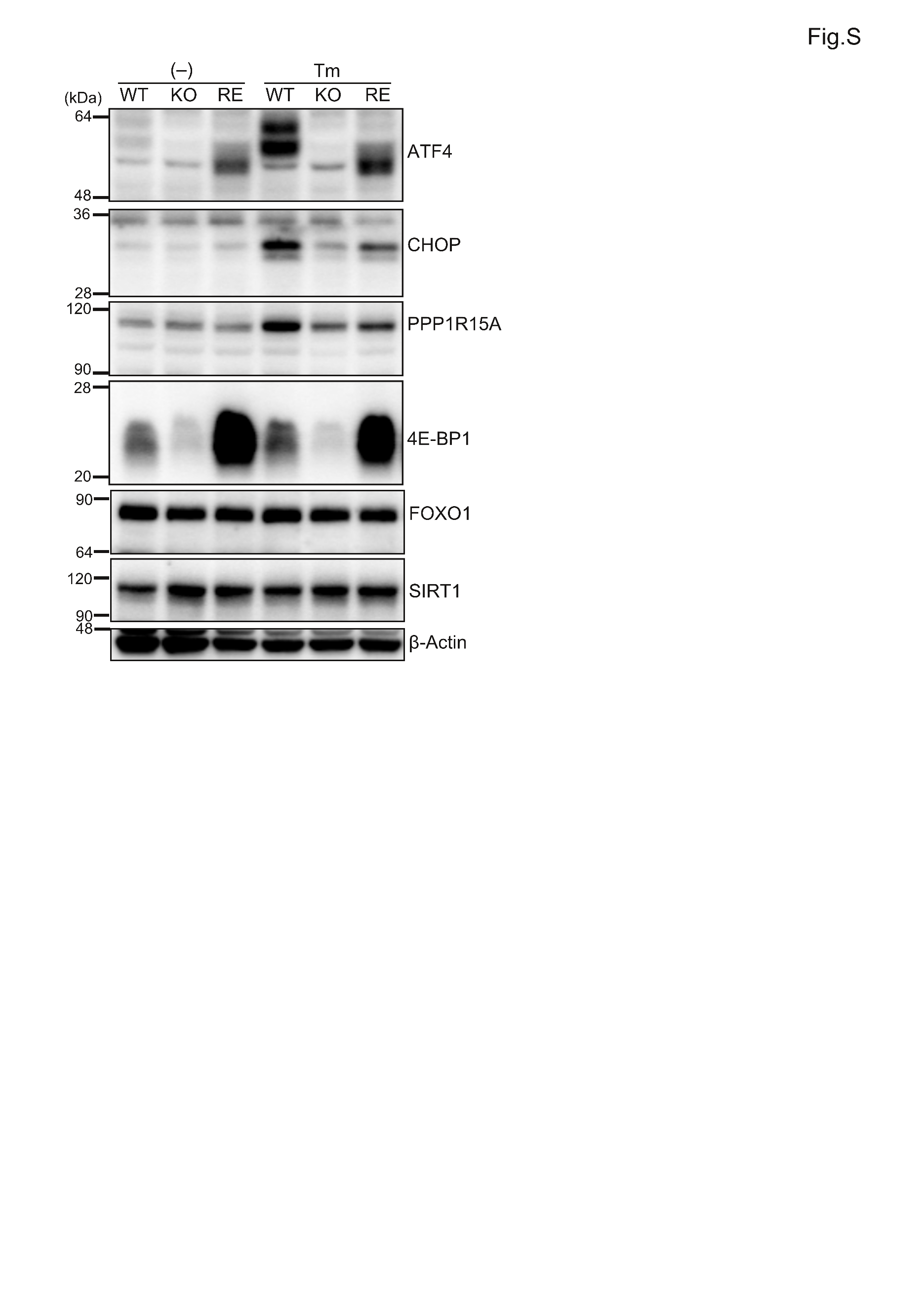
**

**Supplementary Figure 8. Characterization of the *Atf4* knockout CT215 β-cells.**

Representative immunoblots of ATF4, CHOP, PPP1R15A, 4E-BP1, FOXO1 and SIRT1 in CT215 β-cells treated with 0.2 μg/ml tunicamycin (Tm) for 8 h. β-Actin was used as a loading control. WT, wild-type; KO, *Atf4* knockout; RE, KO overexpressing the human ATF4.

**Supplementary Table 1. Primary antibodies used for IHC, immunoblot analyses, and ChIP**

| Name | Application | Dilution | Source |
| --- | --- | --- | --- |
| anti-insulin | IHC | 1:3 | IR00261-2, Agilent Technologies—Dako, Santa Clara, CA |
| anti-glucagon | IHC | 1:2,000 | G2654, Sigma-Aldrich, St. Louis, MO |
| anti-ATF4 | IHC | 1:150 | 11815S, Cell Signaling Technology, Danvers, MA |
| anti-somatostatin | IHC | 1:100 | SC-74556, Santa Cruz Biotechnology, Santa Cruz, CA |
| anti-Ppy | IHC | 1:2,000 | Prepared as reported previously (1). Currently commercially available from Immuno-Biological Laboratories, Fujioka, Japan (#10501) |
| anti-PDX1 | IHC | 1:500 | 06-1379, Merck, Darmstadt, Germany |
| anti-Nkx6.1 | IHC | 1:1,000 | 54551S, Cell Signaling Technology |
| anti-Ki67 | IHC | 1:250 | 12202S, Cell Signaling Technology |
| anti-ALDH1A3 | IHC | 1:200 | NBP2-15339, Novus Biologicals, Centennial, CO |
| anti-P-eIF2α | IB | 1:1,000 | 9721S, Cell Signaling Technology |
| anti-T-eIF2α | IB | 1:1,000 | 5324S, Cell Signaling Technology |
| anti-ATF4 | IB | 1:1,000 | 10835-1-AP, Proteintech, Rosemont, IL |
| anti-CHOP | IB | 1:1,000 | 15204-1-AP, Proteintech |
| anti-4E-BP1 | IB | 1:2,000 | 9644S, Cell Signalling Technology |
| anti-β-actin | IB | 1:3,000 | PM053, Medical & Biological Laboratories, Nagoya, Japan |
| anti-FLAG M2 | IB | 1:1,000 | F1804, Sigma-Aldrich |
| anti-GAPDH | IB | 1:1,000 | M171-3, Medical & Biological Laboratories |
| anti-FoxO1 | IB | 1:1,000 | 2880S, Cell Signaling Technology |
| anti-SIRT1 | IB | 1:1,000 | 07-131, Merck |
| anti-PPP1R15A | IB | 1:3,000 | gift from Dr. David Ron  (Cambridge Institute for Medical Research, Cambridge, UK). |
| anti-ATF4 | ChIP | 1:200 | 11815S, Cell Signaling Technology |
| Normal rabbit IgG | ChIP | 1:200 | 2729S, Cell Signaling Technology |

(1) Hara A, et al. 2019. Development of monoclonal mouse antibodies that specifically recognize pancreatic polypeptide. Endocr J 66:459–468

**Supplementary Table 2. Sequences of primers**

| Name | Application | Sequence (5′–3′) | |  |
| --- | --- | --- | --- | --- |
|  |  | Forward | Reverse | |
| mEif4ebp1 | qPCR | CTAGCCCTACCAGCGATGAG | CCTGGTATGAGGCCTGAATG | |
| mChop | qPCR | GCGACAGAGCCAGAATAACA | GATGCACTTCCTTCTGGAACA | |
| mPpp1r15a | qPCR | TCCTCTAAAAGCTCGGAAGGT | CAAAGCGGCTTCGATCTC | |
| mXbp1s | qPCR | GCTGAGTCCGCAGCAGGT | CAGGGTCCAACTTGTCCAGAAT | |
| mHspa5 | qPCR | CTGAGGCGTATTTGGGAAAG | TCATGACATTCAGTCCAGCAA | |
| mAtf4 | qPCR | ATGATGGCTTGGCCAGTG | CCATTTTCTCCAACATCCAATC | |
| mAtoh8 | qPCR  *in situ* PCR | TCAGCTTCTCCGAGTGTGTG | TAGCCTGTGGCAGGTCACTC | |
| mNanog | qPCR | TCTGCTACTGAGATGCTCTGC | TCCGCATCTTCTGCTTCCTG | |
| mPou5f1 | qPCR | CCCTGCAGAAGGAGCTAGAAC | AGATGGTGGTCTGGCTGAAC | |
| mGapdh | qPCR | TGCGACTTCAACAGCAACTC | CTTGCTCAGTGTCCTTGCTG | |
| mAtf4 floxed | genotyping | CAGGTACAACAAAGATGGGATGTAA | AACCTGAGCTGGTCTATTTCTGAAC | |
| mAtf4 deleted | genotyping | CAGGTACAACAAAGATGGGATGTAA | GTGAGCCCGAGATGATTAAGCTAAG | |
| mAtoh8 | ChIP-qPCR | ATGTTTGCTCTGGGGGCGA | CCAAATCACATTCCACTGGCAC | |
| mWars | ChIP-qPCR | AGGTCATATGGCGGTCAACCTGAA | TCTTCAAGAGCAAGTCCAGCCACT | |
| mAtf3 | ChIP-qPCR | ATTGGTAACCTGGAGTTAAGCGGG | TGAGTGAGACTGTGGCTGGGA | |

**Supplementary Table 3. Key deferentially regulated genes in microarray analysis**

| ID | Gene symbol | Gene name | (F/F+DMSO)  /(KO+DMSO) | | (F/F+Tm)  /(F/F+DMSO) | |
| --- | --- | --- | --- | --- | --- | --- |
|  |  |  | *P*-value | Fold change | *P*-value | Fold change |
| A_52_P453884 | Foxo1 | forkhead box O1 | 0.143 | 0.79 | 0.501 | 1.10 |
| A_51_P138044 | Foxo1 | forkhead box O1 | 0.137 | 0.88 | 0.967 | 1.00 |
| A_55_P2030428 | Sirt1 | sirtuin 1 | 0.467 | 0.83 | 0.800 | 0.94 |
